# Dousing the flame: reviewing the mechanisms of inflammatory programming during stress-induced intrauterine growth restriction and the potential for ω-3 polyunsaturated fatty acid intervention

**DOI:** 10.3389/fphys.2023.1250134

**Published:** 2023-09-01

**Authors:** Melanie R. White, Dustin T. Yates

**Affiliations:** Stress Physiology Laboratory, Department of Animal Science, University of Nebraska-Lincoln, Lincoln, NE, United States

**Keywords:** adaptive fetal programming, developmental origins of health and disease, DOHAD, fetal growth restriction, intrauterine growth restriction, IUGR, low birthweight, metabolic programming

## Abstract

Intrauterine growth restriction (IUGR) arises when maternal stressors coincide with peak placental development, leading to placental insufficiency. When the expanding nutrient demands of the growing fetus subsequently exceed the capacity of the stunted placenta, fetal hypoxemia and hypoglycemia result. Poor fetal nutrient status stimulates greater release of inflammatory cytokines and catecholamines, which in turn lead to thrifty growth and metabolic programming that benefits fetal survival but is maladaptive after birth. Specifically, some IUGR fetal tissues develop enriched expression of inflammatory cytokine receptors and other signaling cascade components, which increases inflammatory sensitivity even when circulating inflammatory cytokines are no longer elevated after birth. Recent evidence indicates that greater inflammatory tone contributes to deficits in skeletal muscle growth and metabolism that are characteristic of IUGR offspring. These deficits underlie the metabolic dysfunction that markedly increases risk for metabolic diseases in IUGR-born individuals. The same programming mechanisms yield reduced metabolic efficiency, poor body composition, and inferior carcass quality in IUGR-born livestock. The ω-3 polyunsaturated fatty acids (PUFA) are diet-derived nutraceuticals with anti-inflammatory effects that have been used to improve conditions of chronic systemic inflammation, including intrauterine stress. In this review, we highlight the role of sustained systemic inflammation in the development of IUGR pathologies. We then discuss the potential for ω-3 PUFA supplementation to improve inflammation-mediated growth and metabolic deficits in IUGR offspring, along with potential barriers that must be considered when developing a supplementation strategy.

## 1 Introduction

Fetal intrauterine growth restriction (IUGR) occurs in an estimated 1 out of every 5 pregnancies worldwide, resulting in almost 30 million babies being affected by the condition each year (Kesavan and Devaskar, 2019). Asymmetrical IUGR is a pathological cause of low birth weight and small-for-gestational-age (SGA) newborns. Unlike genetic causes of SGA, IUGR is an adaptive response to chronic fetal hypoxemia, hypoglycemia, and other sustained nutritional stresses, since slower fetal growth rates require less nutrients (Sharma et al., 2016; Kesavan and Devaskar, 2019). However, these adaptations also reduce newborn vigor and substantially increases the risk for early morbidity and mortality (Garite et al., 2004). Globally, IUGR-born infants suffer 3-fold greater perinatal demise than babies born at appropriate size for their gestational age (Garite et al., 2004). Most commonly, IUGR-born babies survive but are at much greater risk for chronic metabolic disorders, including obesity, hyperlipidemia, and type 2 diabetes (Hales and Barker, 2001; Hicks and Yates, 2021). This is because the programmed metabolic thrift that underlies IUGR and benefits the nutrient-deprived fetus becomes an environmental mismatch after birth, as offspring are no longer hypoxemic and typically have better nutritional opportunities (Gibbs et al., 2020; Posont et al., 2021). Hales and Barker were the first to link IUGR-induced low birthweight to lifelong metabolic dysfunction and the myriad associated health disorders (Hales et al., 1991; Hales and Barker, 1992). In the decades since, research has begun to identify the root causes for this link. For example, IUGR-born infants typically undergo compensatory catch-up growth prior to adolescence that is driven more so by fat deposition than by lean tissue growth (Ong et al., 2000; Dulloo et al., 2012). Greater adiposity compounds programmed metabolic thrift and further increases the risk for insulin resistance, high blood pressure, and greater body mass index (Ong and Dunger, 2000; Mericq et al., 2005; Lurbe et al., 2009). In adulthood, these risks often manifest in diabetes, hypertension, central obesity, hyperlipidemia, and heart disease (Barker et al., 1993; Barker et al., 2002). Although modern medical advances have markedly improved global IUGR infant survival rates, prevalence of the condition has remained static for decades (Goldenberg and Culhane, 2007; Mathews and Driscoll, 2017). Developing countries in South Asia and Sub-Saharan Africa remain at the highest risk for IUGR pregnancies, but rates in developed countries have also increased since 1981 (Gurung et al., 2022). Moreover, rates among African-Americans and among individuals from low-income areas have increased disproportionately in the US (Goldenberg and Culhane, 2007).

Prenatal stressors in livestock and mammalian wildlife species induce the same fetal programming mechanisms responsible for IUGR in humans (De Blasio et al., 2007a; Yates et al., 2019). The lack of perinatal vigor makes IUGR-born animals more susceptible to starvation and predation, which is a major animal welfare issue that costs the US livestock industry on average about 8% of its annual product (Milligan BNF and Kramer, 2002; Wu et al., 2006). Like humans, most IUGR-born animals survive but exhibit inefficient growth and less desirable carcasses that create a great economic burden (Greenwood et al., 1998; Ogata et al., 1999; Milligan BNF and Kramer, 2002). There is an explicitly-recognized need for more efficient livestock production to feed the world’s rapidly increasing population, which is expected to double by 2050 (Godfray et al., 2010). Improving growth efficiency in IUGR-born livestock would allow more food to be produced from the same number of animals without overgrowing normal animals. The clear impact of IUGR on lifelong health in humans and on sustainability for the livestock industry makes strategies to target maladaptive fetal programming a fundamental need. This review presents the evidence for the role of inflammatory programming in IUGR-associated metabolic pathologies and discusses the potential nutraceutical efficacy of anti-inflammatory omega-3 polyunsaturated fatty acids (ω-3 PUFA) in mitigating those outcomes.

## 2 Characteristics of IUGR

### 2.1 Placental insufficiency: the common culprit for IUGR

Fetal IUGR can result from any maternofetal stressor that stunts placental development or otherwise limits fetal nutrient supply. For humans, environmental and social stresses, nutritional imbalance due to poor or limited diet, unhealthy lifestyle choices, and pregnancies occurring after the age of 35 are some of the many factors that increase the risk of impaired placental development and growth (Beard et al., 2009; Nardozza et al., 2017). For livestock, common causes of fetal and placental growth restriction include chronic heat stress, restricted feed intake due to drought or mismanagement, and grazing of noxious forages (Greenwood and Cafe, 2007; Robinson et al., 2013). Placental insufficiency is also common in small ruminants carrying multi-fetal pregnancies and in swine carrying large litters (Milligan BNF and Kramer, 2002; De Blasio et al., 2007a). Inadequate blood flow and/or nutrient supply during the critical window for placental development (mid-gestation for most species) causes stunting that ultimately prevents the placenta from fulfilling the expanding nutritional needs of the growing fetus, even when the cause of stunting has been resolved (Burton and Jauniaux, 2018). As the fetus outgrows the stunted placenta in late gestation, progressive IUGR ensues in a predictable pattern (Nardozza et al., 2017). Several animal models have been developed to study IUGR, from pigs (Tang and Xiong, 2022) to non-human primates (Chassen et al., 2020). Sheep are particularly good for IUGR research. The clinical characteristics and developmental milestones of sheep pregnancies are remarkably comparable to humans and other ruminant livestock, as previously described in detail (Yates et al., 2018; Beede et al., 2019). Sheep fetuses are uncommonly resilient, and ewes are typically easy to obtain and house (Beede et al., 2019). A popular model for natural IUGR induction is to expose pregnant ewes to chronic heat stress during peak placental growth, which reliably induces placental insufficiency and in turn fetal IUGR (Beede et al., 2019). Other natural IUGR sheep models include maternal nutrient restriction, maternofetal inflammation, high-altitude hypoxemia, and behavioral stress (Zhang et al., 1998; Beede et al., 2019). Placental insufficiency can also be created artificially via placental embolization, umbilical artery ligation, or carunclectomy (Beede et al., 2019).

Placental stunting is the result of stress-induced alterations in maternal nutrient flux. Normally, robust repartitioning of maternal nutrients occurs to support the gravid uterus, which is facilitated in part by greater uterine blood flow (Thaler et al., 1990). Chronic stress reduces maternal nutrient repartitioning to the uterus by slowing uterine blood flow up to 50% in sheep models (Thureen et al., 1992; Lang et al., 2000; Wallace et al., 2008). Reduced uterine O_2_ delivery is particularly damaging, as a rodent model of maternal hypoxemia yielded placental and fetal IUGR despite a compensatory increase in uterine blood flow (Lane et al., 2020). Placental vasculature expands rapidly beginning about 0.3 of pregnancy, and stress-induced suppression of vasculogenesis during this critical window cannot be recovered later in pregnancy (Burton and Jauniaux, 2018). Underdeveloped placental villi and poor fetoplacental angiogenesis in IUGR pregnancies culminate in as much as two-fold reductions of placentome volume and maternofetal vascular interface (Mayhew et al., 2004; Edwards et al., 2020). Indeed, reduced peripheral capillary and villous surface areas are hallmarks of the IUGR placenta (Teasdale, 1984). Not surprisingly, two key angiogenic factors are dysregulated in IUGR placental tissues: vascular endothelial growth factor (VEGF) and placental growth factor (PlGF) (Regnault et al., 2002; Regnault et al., 2003). Diminished maternofetal interface slows the movement of O_2_, glucose, and other molecules that cross the placenta via simple or facilitated diffusion (Regnault et al., 2003). Studies in IUGR sheep indicate a 50%–70% disparity in maternofetal O_2_ gradients (Limesand et al., 2007; Beer et al., 2021). Nutrients that cross via facilitated diffusion or active transport are also slowed by reduced placental expression of transporters. For example, sheep pregnancies made IUGR by maternal overfeeding or heat stress had reduced placental glucose transport (Wallace et al., 2003; Brown et al., 2015) that coincided with less of the Glut1, Glut3, and Glut8 glucose transporters (Wallace et al., 2005). Placental transport of amino acids was also reduced in IUGR sheep pregnancies (Brown et al., 2012). In humans and rodents, this coincided with downregulation of the Na^+^-dependent neutral amino acid transporter A system, which moves alanine, serine, glutamine and glycine (Jansson et al., 2006; Shibata et al., 2008; Alkhalefah et al., 2021). Conversely, placental fatty acid transporters were downregulated in IUGR mice but increased in the placenta of nutrient-restricted baboons and in human pregnancies complicated by IUGR (Assumpcao et al., 2017; Xu et al., 2019; Chassen et al., 2020).

### 2.2 The hallmark IUGR fetal phenotype

#### 2.2.1 Fetal pathophysiology

Stress-stunted placentas can typically fulfill the relatively modest fetal nutrient demands in early and mid-gestation, but exponential fetal growth during late gestation pushes O_2_ and nutrient requirements beyond the capacity of the stunted placenta (Limesand et al., 2013; Macko et al., 2013). As the fetus continues to grow, nutrient deficits progressively worsen. Natural sheep models for IUGR produce up to 50% reductions in fetal blood glucose near term (Saker et al., 1999; Limesand et al., 2007; Yates et al., 2016; Edwards et al., 2020). Endogenous hepatic glucose production is engaged in the IUGR fetus but only partially compensates for its hypoglycemia (Fowden and Silver, 1995; Limesand et al., 2007; Thorn et al., 2009; Thorn et al., 2013). In response to limited nutrient availability, the fetus engages its own nutrient repartitioning adaptations that prioritize vital nervous and endocrine tissues over others, particularly skeletal muscle (Poudel et al., 2015). Greater circulating lactate concentrations observed when IUGR fetal sheep were experimentally made hyperglycemic were consistent with a shift in muscle glucose metabolism from oxidative phosphorylation to anerobic glycolysis (Thorn et al., 2013; Lacey et al., 2021). Such shift coincides with and is perhaps necessitated by the hypoxemic state of the fetus (Regnault et al., 2003; Brown et al., 2015). However, less oxidative phosphorylation diminishes energy status by reducing production of ATP (Cree-Green et al., 2018). Despite this, IUGR fetuses exhibited greater circulating CO_2_ due to compromised placental gas transfer, which can affect acid-base balance (Macko et al., 2013; Lacey et al., 2021). Disruptions in lipid homeostasis included 10%–25% lower circulating cholesterol, which were reported in IUGR human and rodent fetuses at term despite markedly higher precursor concentrations and slower clearance rates (Cadaret et al., 2019a; Pecks et al., 2019). This indicates that deficits were due at least partially to impaired *de novo* cholesterol synthesis by the IUGR fetal liver rather than solely due to deficient placental transport. Conversely, ∼15% reductions in circulating triglycerides coincided with less placental fatty acid transporter expression and greater disparity in maternofetal triglyceride gradients in IUGR fetal models (Meyer et al., 2010; Cadaret et al., 2019a; Xu et al., 2019). Diminished placental amino acid transporter expression together with less effective Na^+^/K^+^-ATPase support is reflected in circulating amino acid profiles in IUGR fetuses (Stremming et al., 2020; Rosario et al., 2021). Brown et al. (2012) observed reduced placental transport of isoleucine, leucine, phenylalanine, tryptophan, methionine, and tyrosine, which notably lowered fetal circulating arginine and methionine and muscle protein synthesis patterns in IUGR fetal sheep. Interestingly, concentrations of some amino acids were actually increased in these fetuses, which was presumably a byproduct of reduced protein synthesis (Armstrong, 1973). Even electrolyte homeostasis can be disrupted by placental insufficiency, as fetal blood concentrations of Na^+^, K^+^, Ca^++^, and Cl^−^ were increased in IUGR fetal sheep (Jansson et al., 2006; Bacchetta et al., 2009; Beer et al., 2021; Lacey et al., 2021).

Nutrient and O_2_ paucities stimulate fetal stress responses that include systemic inflammatory and adrenergic components (Yates et al., 2012a; Berbets et al., 2021). Hypoxemia, and to a lesser extent hypoglycemia, stimulate secretion of the catecholamines norepinephrine and epinephrine from the fetal adrenal medulla (Gardner et al., 2002; Ly et al., 2011; Yates et al., 2012a). Circulating norepinephrine (the primary fetal adrenal catecholamine) was elevated by as much as 8-fold in IUGR fetal sheep, and hypercatecholaminemia was among the earliest indicators of placental insufficiency (Macko et al., 2013; Chang et al., 2019a). Catecholamines are most associated with physiological mechanisms aimed at immediate survival, and sustained exposure of tissues can disrupt β adrenergic programming, the details and implications of which have been reviewed elsewhere (Yates et al., 2011; Posont et al., 2017; Gibbs and Yates, 2021). Perhaps the most consequential effect of fetal hypercatecholaminemia is suppressed insulin activity. Basal circulating insulin concentrations were often modestly decreased in IUGR fetal sheep, but glucose-stimulated insulin secretion was almost completely suppressed (Leos et al., 2010; Macko et al., 2013; Cadaret et al., 2019b). In contrast, maternal nutrient restriction-induced IUGR did not affect insulin concentrations (Edwards et al., 2020), which indicates that hypoxemia and hypercatecholaminemia are the suppressors of insulin secretion. Not surprisingly, circulating IGF-1 concentrations were likewise reduced in IUGR fetal sheep during mid- and late-gestation (Thorn et al., 2009; Macko et al., 2013; Rozance et al., 2018). As hypoxemia stimulates the adrenal medulla, hypoglycemic conditions stimulate the adrenal cortex to secrete cortisol, a steroid stress hormone associated with changes in intermediary metabolism (Thorn et al., 2013; Jonker et al., 2018). This has been documented across many IUGR models and among several species (Sutherland et al., 2012; Li et al., 2013; Thorn et al., 2013), and it may ultimately lead to reduced sensitivity of the cortisol axis (Iwata et al., 2019). Despite the anti-inflammatory effects of cortisol, IUGR fetuses exhibited elevated circulating inflammatory cytokines, including tumor necrosis factor α (TNFα)*,* interleukin-1β (IL-1β), and IL-6 (Cadaret et al., 2019b; Zhang et al., 2021), and lower concentrations of anti-inflammatory IL-10 and IL-12 (Huang et al., 2019). Cytokines mediate inflammatory responses to pathogens, reactive oxygen species, and toxins, but they are also responsive to hypoxemia and other physiological stressors (Lacy and Stow, 2011; Hicks and Yates, 2021). They are released in greatest volume from circulating white blood cells and resident tissue macrophages and mast cells, and they act on tissues throughout the body (Tracey and Cerami, 1993; Lacy and Stow, 2011). Elevated inflammatory cytokines in IUGR fetuses can increase catabolism of skeletal muscle and affect mobilization of nutrient stores (Hicks and Yates, 2021). The concurrent appearance of systemic inflammation and hypercatecholaminemia in near-term IUGR fetuses is somewhat paradoxical, as macrophagic release of TNFα and IL-6 is suppressed by adrenergic stimulation under normal conditions (Donnelly et al., 2010; Papandreou et al., 2016). Nonetheless, the combined heightened inflammatory and adrenergic tones mediate many of the changes in metabolism and growth that IUGR fetuses exhibit (Macko et al., 2013; Hicks and Yates, 2021).

#### 2.2.2 Fetal growth restriction

The bodyweights of IUGR fetal sheep and pigs were reduced by as much as 55% near term (Tree et al., 2016; Cadaret et al., 2019c; Tang and Xiong, 2022), and IUGR-born offspring remained lighter well into the neonatal period (Gibbs et al., 2020; Shoji et al., 2020). However, growth restriction is not equivalent among all fetal tissues. In fact, sustained nutrient insufficiency and the resulting fetal stress response yields hallmark asymmetric growth by disproportionally slowing muscle accretion relative to cranial and skeletal growth (Lapillonne et al., 1997). This was reflected in reduced muscle protein accretion rates, muscle morphometrics, and body length-to-mass ratios of IUGR fetal sheep (Yates et al., 2016; Rozance et al., 2018; Cadaret et al., 2019b). In human fetuses for whom adiposity is naturally high, disproportionally slower fat deposition was also apparent from ultrasound diagnosis of IUGR (Padoan et al., 2004; Ikenoue et al., 2021). The fetal hypoxemia-hypercatecholaminemia-hypoinsulinemia cascade appears instrumental in muscle growth restriction, as experimental induction of each factor individually produced some degree of IUGR in sheep (Philipps et al., 1991; Bassett and Hanson, 1998; Rozance et al., 2018). Hypoxemia-induced inflammation also limited muscle mass by increasing protein catabolism, reducing protein accretion, and altering muscle stem cell function (Li W. et al., 2009; Wang et al., 2014; Madaro et al., 2018; Posont et al., 2022). Less severe reductions in body length, head circumference, and cannon bone length observed in IUGR fetal lambs reflected the modest nature of structural growth restriction (Rozance et al., 2018; Cadaret et al., 2019c; Sandoval et al., 2020). As with bodyweight, however, these mild deficits in structural metrics persisted postnatal (Gibbs et al., 2020; Shoji et al., 2020). Ultimately, most IUGR-born offspring undergo a period of catch-up growth that diminishes or eliminates their weight disparity (Dulloo et al., 2006). However, this compensatory weight gain results from greater fat accumulation and does not reflect recovery of muscle mass (Ong et al., 2000; Gibbs et al., 2020). Consequently, IUGR-born adolescents and adults are more likely to develop high body mass indices (Fagerberg et al., 2004; Zinkhan et al., 2018). For humans, altered body composition was associated with a higher risk for metabolic and cardiovascular dysfunction later in life (Ong et al., 2000; Fagerberg et al., 2004). In food animals, IUGR-altered body composition resulted in smaller and less valuable carcasses (Greenwood et al., 2005; Greenwood and Bell, 2019).

Preferential reappropriation of fetal nutrients from muscle and other peripheral soft tissues to vital brain and endocrine tissues is reflected in blood flow patterns. In IUGR fetal sheep, blood flow was maintained or increased in every region of the brain, and pancreatic and adrenal blood flow increased by up to 2-fold (Poudel et al., 2015). Conversely, blood flow to the hindlimb, which is about 45% skeletal muscle (Hicks et al., 2021), was reduced by half (Poudel et al., 2015; Hicks et al., 2021). In a baboon model of IUGR, less hindlimb blood flow was associated with decreased size and distensibility of the external iliac and femoral arteries, which was sustained into adulthood (Kuo et al., 2018). In contrast, lower ultrasound-estimated resistance in the carotid artery of human IUGR fetuses reflected the brain-sparing characteristic of asymmetric growth restriction (Groenenberg et al., 1989). Preferential delivery of blood and nutrients maintained brain weights across sheep models for IUGR (Yates et al., 2016; Rozance et al., 2018; Wallace et al., 2020; Posont et al., 2021) even when heart, lung, and liver weights were reduced (Hyatt et al., 2007; Cadaret et al., 2019b; Wallace et al., 2020). Not surprisingly, weights of individual hindlimb muscles relative to fetal weight or hindlimb length were reduced in IUGR fetal sheep by up to 40% (Rozance et al., 2018; Lacey et al., 2021).

Impaired myoblast function and slower protein synthesis each contributed to reduced IUGR muscle mass (Yates et al., 2014; Soto et al., 2017; Rozance et al., 2018; Felicioni et al., 2020), which persisted after birth (De Blasio et al., 2007a; Gosby et al., 2009; Gibbs et al., 2020). Myoblasts are the myogenic stem cells that facilitate muscle fiber hypertrophy in late gestation and after birth when fiber numbers have become largely static (Maier et al., 1992; Wilson et al., 1992). Myoblasts undergo rate-limiting proliferation and differentiation steps before fusing with existing muscle fibers to increase the myonuclear content/protein capacity of the fiber (Allen et al., 1979; Yates et al., 2012b). Histological and *ex vivo* assessments indicated that differentiation capacity was impaired in myoblasts from IUGR fetal lambs (Yates et al., 2014; Yates et al., 2016; Soto et al., 2017; Posont et al., 2022). Myoblast proliferation was also typically reduced by IUGR intrauterine conditions, although proliferation was increased in one model induced by maternofetal inflammation (Soto et al., 2017; Cadaret et al., 2019a; Posont et al., 2022). Myoblast dysfunction is reflected in expression of key myogenic transcription factors, which hint at mechanisms for IUGR myoblast deficits. Specifically, differentiation factors myoD and myogenin were reduced in IUGR fetal myoblasts near term, whether assessed *in vivo* or *ex vivo* (Yates et al., 2014; Chang et al., 2019b; Rozance et al., 2021; Posont et al., 2022). Reduced myoblast proliferation, differentiation, and fusion slowed myonuclear accumulation in all muscle fiber types, which in IUGR fetal lambs restricted cross-sectional fiber area by as much as 60% (Yates et al., 2016; Chang et al., 2019b). Smaller muscle fibers have also been documented in IUGR piglets and rats (Cadaret et al., 2019a; Felicioni et al., 2020). Although some variation exists among IUGR models, species, and even specific muscles, disproportional reduction of muscle and other soft tissues as a means of sparing brain and structural growth is a well-conserved phenotype. Table 1 illustrates the distinct size reductions of specific tissues and organs that yield asymmetric body composition.

**TABLE 1:** Summary of literature reporting stress-induced placental and fetal growth restriction.

Age	Species	Placental weight	Body weight	Organ/Muscle weight		Study
*Spontaneous IUGR*						
Newborns	Humans		↓34%			Krajewski et al. (2014)
			↓33%			Miranda et al. (2018)
		↓69%	↓80%			Paolini et al. (2001)
			↓34%			Pecks et al. (2019)
			↓13%			Sharma et al. (2007)
			↓12%			Xiao and Li (2005)
		↓12%	↓14%			Souza et al. (2016)
*Maternal Nutrient Restriction*						
Fetus	Baboon	↓10%	↓7%	Brain, ND		Chassen et al. (2020)
	Mouse	↓38%	↓48%			Ganguly et al. (2016)
	Rat	↓12%	↓21%			Jansson et al. (2006)
			↓26%	Brain, ND	Liver, ↓26%	Sadiq et al. (1999)
		↓9%	↓15%	Brain, ND	Kidney, ND	Alkhalefah et al. (2021)
				Liver, ND	Heart, ND	
	Sheep		↓0%–32%	Brain, ↓0%–9%	Kidney, ↓0%–24%	Sandoval et al. (2020)
				Liver, ↓11%–43%	Heart, ↓0%–22%	
				*Gastrocnemius*, ↓0%–30%	*Soleus*, ↓0%–33%	
				*L. dorsi*, ↓0%–38%	Fiber CSA, ↓0%–31%	
			↓21%	Liver, ↓15%		Zhang et al. (2021)
Neonate	Baboon		↓10%	Heart, ND		Kuo et al. (2018)
	Rat		↓11%			Chen et al. (2016)
			ND	Brain, ND	Liver, ND	Sadiq et al. (1999)
			↓14%			Desai et al. (2009)
			↓11%	Liver, ↓14%		He et al. (2018)
			↓4%			Gosby et al. (2009)
			↓12%			Tree et al. (2016)
			↓36%			Xing et al. (2019)
	Mouse		↓8%			Chisaka et al. (2015)
*Maternal Overnutrition*						
Fetus	Sheep	↓46%	↓34%	Brain, ↓9%	Liver, ↓36%	Wallace et al. (2003)
			↓26%			Wallace et al. (2008)
Neonate	Sheep		↓33%			Wallace et al. (2008)
		↓43%	↓42%			Wallace et al. (2020)
*Maternofetal Inflammation*						
Fetus	Rat		↓8%	Hindlimb CSA, ↓14%		Cadaret et al. (2019a)
	Sheep		↓22%	Brain, ND	Kidney, ↓23%	Cadaret et al. (2019b)
				Liver, ↓8%	Lungs, ↓9%	
				Heart, ↓8%		
Neonate	Sheep		↓18%	Brain, ND	Kidney, ↓16%	Posont et al. (2021)
				Liver, ↓9%	Lungs, ↓14%	
				Heart, ↓24%		
*Maternal Heat Stress*						
Fetus	Sheep	↓55%	↓43%	Brain, ↓14%	Liver, ↓47%	Brown et al. (2012)
				Carcass, ↓48%		
		↓47%	↓40%			Brown et al. (2015)
			↓40%	*Biceps femoris*, ↓45%	*Tibialis anterior*, ↓48%	Chang et al. (2019a)
				*Flexor dig. superficialis*, ↓48%	Fiber CSA, ↓37%	
				Fiber no., ↓32%		
		↓36%	↓36%	Brain, ↓11%	Kidney, ↓29%	Chang et al. (2021)
				Liver, ↓27%	Lungs, ↓34%	
				Heart, ↓28%	Pancreas, ↓21%	
				Spleen, ↓32%	*Tibialis anterior*, ↓35%	
				*Flexor dig. superficialis*, ↓33%	*Gastrocnemius*, ↓37%	
				*Extensor dig. longus*, ↓57%	*Soleus*, ↓58%	
				Fiber CSA, ↓36%	Fiber no., ↓40%	
			↓26%	Brain, ↓9%	Kidney, ↓24%	Lacey et al. (2021)
				Liver, ↓9%	Lungs, ↓25%	
				Heart, ↓22%	Hindlimb, ↓23%	
				*Semitendinosus*, ↓26%	*Soleus*, ↓42%	
				*Flexor dig. superficialis*, ↓36%	*L. dorsi*, ↓25%	
			↓58%	Pancreas, ↓59%		Limesand et al. (2005)
		↓60%	↓55%			Limesand et al. (2006)
		↓40%	↓15%	Brain, ND	Liver, ↓22%	Limesand et al. (2013)
				Pancreas, ↓8%		
		↓38%	↓3%	Brain, ND	Liver, ND	Macko et al. (2013)
		↓68%	↓53%	Brain, ↓15%	*Semitendinosus*, ↓51%	Pendleton et al. (2019)
				*Biceps femoris*, ↓50%		
*Maternal Heat Stress (Cont’d)*						
Fetus	Sheep	↓51%	↓42%			Regnault et al. (2003)
		↓54%	↓56%	Pancreas, ↓42%		Rozance et al. (2015)
		↓42%	↓41%	Brain, ↓14%	Hindlimb, ↓46%	Rozance et al. (2018)
				*Biceps femoris*, ↓45%	*Gastrocnemius*., ↓45%	
				*Flexor dig. superficialis*, ↓48%	*Tibialis anterior*, ↓48%	
				*Extensor dig. longus*, ↓45%		
			↓36%	*Bicpes femoris*, ↓32%	*Gastrocnemius*., ↓32%	Soto et al. (2017)
				*Tibialis anterior*, ↓23%	*Soleus*, ↓25%	
		↓60%	↓56%	Liver, ↓62%		Thorn et al. (2009)
		↓41%	↓38%			Thorn et al. (2013)
		↓53%	↓35%	Liver, ↓43%	Heart, ↓38%	Wai et al. (2018)
				Kidney, ↓47%	Pancreas, ↓14%	
				Spleen, ↓43%	*Biceps femoris*, ↓34%	
				*Flexor dig. superficialis*, ↓48%	*Tibialis anterior*, ↓35%	
				*Extensor dig. longus*, ↓33%	*Gastrocnemius*, ↓37%	
		↓70%	↓68%			Yates et al. (2014)
		↓56%	↓55%			Yates et al. (2016)
Neonate	Sheep	↓50%	↓21%			Chen et al. (2010)
			↓33%			Yates et al. (2019)
			↓21%	Brain, ND	Liver, ND	Cadaret et al. (2022)
				Heart, ↓37%	Lungs, ↓9%	
				Kidney, ↓13%	Hindlimb, ↓32%	
				*Flexor dig. superficialis*, ↓19%		
Juvenile	Sheep		↓12%	Brain, ND	Liver, ↓13%	Gibbs et al. (2021)
				Heart, ↓29%	Lungs, ↓29%	
				Kidney, ↓15%	Hindlimb, ↓17%	
				*Flexor dig. superficialis*, ↓22%	*L. dorsi* CSA, ↓14%	
				Fiber CSA, ↓21%		
*Carunclectomy*						
Fetus	Sheep	↓65%	↓33%	Brain, ↓9%	Liver, ↓51%	Poudel et al. (2015)
				Heart, ↓36%	Kidney, ↓29%	
				Lungs, ↓40%	Pancreas, ↓32%	
				Spleen, ↓52%		
Neonate	Sheep		↓26%	Fat, ND	Adipocyte CSA, ND	Duffield et al. (2009)
		↓39%	↓25%	Fat, ↑40%	*Semitendinosus*, ↓18%	De Blasio et al. (2007a)
				*Biceps femoris*, ND	*Flexor capri*, ↓49%	
				*Gastrocnemius*, ↓18%	*Tibialis anterior*, ↓32%	
				*Soleus*, ND	*Vastus lateralis*, ↓30%	
				*Extensor dig. longus*, ND		
Juvenile	Sheep		ND	Fat, ↑49%		De Blasio et al. (2007b)
*Uterine Artery Ligation*						
Fetus	Rat		↓26%	Brain, ND	Liver, ↓26%	Sadiq et al. (1999)
	Sheep	↓27%–34%	↓15%–33%	Brain, ↓0%–6%	Liver, ↓28%–36%	Lang et al. (2000)
				Heart, ↓16%–31%	Lungs, ↓11%–27%	
			↓16%	Lungs, ↓10%		Sutherland et al. (2012)
Neonate	Rat		ND	Brain, ND	Liver, ↓7%	Sadiq et al. (1999)
*Uterine Overcrowding*						
Neonate	Pig		↓40%	Brain, ↓12%	Liver, ↓45%	Felicioni et al. (2020)
				Heart, ↓36%	*Semitendinosus*, ↓52%	
				*Semitendinosus* CSA, ↓14%	Fiber CSA, ↓16%	
				Fiber no., ↓32%		
			↓47%			Niu et al. (2019)

CSA, cross-sectional area; ND, not different.

#### 2.2.3 Fetal metabolic adaptations: muscle glucose metabolism

Limiting skeletal muscle growth contributes to fetal nutrient sparing necessitated by placental insufficiency (Brown, 2014). Additionally, the IUGR fetus alters tissue-specific utilization, metabolism, and storage of nutrients (Rozance and Wolfsdorf, 2019; Yates et al., 2019; Zhang et al., 2021). As with growth restriction, these changes disproportionally affect muscle. Several IUGR fetal sheep models found that muscle-specific glucose oxidation rates were reduced by up to 80% near term, which along with reduced hepatic oxidative metabolism produced a ∼50% reduction in whole-fetus glucose oxidation (Peterside et al., 2003; Limesand et al., 2007; Brown et al., 2015; Cadaret et al., 2019b). This deficit was observed under normal resting conditions and experimental hyperinsulinemia, and it persisted well after birth (Gibbs et al., 2021; Posont et al., 2021; Cadaret et al., 2022). Impaired glucose oxidation also occurred despite normal rates of glucose uptake by muscle, which has been observed before (Rozance et al., 2018; Cadaret et al., 2019c) and after birth (Gibbs et al., 2021; Posont et al., 2021; Cadaret et al., 2022). Although IUGR muscle expressed less of the insulin-dependent glucose transporter Glut4 in some studies (De Blasio et al., 2012; Duan et al., 2016; Yates et al., 2019; Jones et al., 2022), glucose uptake may have been rescued by an increase in the insulin-independent transporter Glut1 (Brown et al., 2015; Yates et al., 2019). Moreover, it should be noted that some studies found no changes in IUGR muscle content of Glut1 or Glut4 (Limesand et al., 2007; Garg et al., 2009). Not surprisingly, Glut1 expression in the brain was increased by over 60% in IUGR fetal lambs and rats as well as in IUGR-born neonatal rats (Sadiq et al., 1999; Limesand et al., 2007), which is consistent with brain sparing during chronic hypoglycemia (Gibbs and Yates, 2021).

Reduced skeletal muscle glucose oxidation rates coincided with lower proportions of slow oxidative (type I) fibers relative to intermediate (type IIa) and fast glycolytic (type IIx) fibers in hindlimb muscles of IUGR fetal sheep and loin muscles of IUGR fetal pigs (Wang et al., 2013; Yates et al., 2016; Stremming et al., 2022). Furthermore, IUGR muscle exhibited reduced activity of pyruvate dehydrogenase and citrate synthase enzymes that generate Krebs cycle intermediates, as well as increased expression of (inhibitory) pyruvate dehydrogenase kinase and impaired function of Electron Transport Chain Complex I (Brown et al., 2015; Pendleton et al., 2019; Stremming et al., 2022). These observations, combined with reduced O_2_ utilization, normal glucose utilization, and greater lactate dehydrogenase B content (Brown et al., 2015; Pendleton et al., 2019), indicated that much of the reduction in glucose oxidative phosphorylation was replaced by greater glycolytic lactate production. This is evident in elevated circulating lactate concentrations, which were as much as 3-fold greater in IUGR fetuses, particularly during experimental hyperglycemia or hyperinsulinemia (Limesand et al., 2007; Davis et al., 2021; Camacho et al., 2022). Lactate-O_2_ quotient was also 2-fold greater, meaning that more lactate was produced from each mole of O_2_ consumed (Rozance et al., 2018). Like other IUGR pathologies, hyperlactatemia appears to be driven primarily by hypoxemia-induced hypercatecholaminemia. In fetal sheep made experimentally hypoxemic (but not hypoglycemic) for 9 days, the 3-fold increase in circulating norepinephrine resulted in 20% less glucose oxidation, 3.5-fold greater circulating lactate, and 2-fold greater fetal lactate production (Jones et al., 2022). This model also demonstrated that hyperlactatemia may indicate condition severity, as circulating lactate concentrations were elevated by robust fetal hypercatecholaminemia (3-fold or greater increase) but not by a more modest 1.4-fold increase in norepinephrine (Jones et al., 2019; Rozance et al., 2021; Jones et al., 2022). Catecholamine-induced hyperlactatemia also persisted in IUGR-born neonates (Chen et al., 2010). Some studies have reported normal blood lactate in IUGR fetuses (Wai et al., 2018; Cadaret et al., 2019b; Pendleton et al., 2020), but this does not necessarily mean that lactate was produced at normal rates. Rather, IUGR fetuses were shown to engage hepatic gluconeogenesis that converts lactate and other substrates to glucose, a mechanism that is largely idle under normal intrauterine conditions (Limesand et al., 2007). This was reflected in elevated expression of gluconeogenic enzymes such as phosphoenolpyruvate carboxykinase and glucose 6-phosphatase and their transcriptional promoters in IUGR sheep, pigs, and rodents in late gestation and after birth (Peterside et al., 2003; Vuguin et al., 2004; Limesand et al., 2007; Thorn et al., 2009; Brown et al., 2015; Ying et al., 2017). Hepatic gluconeogenesis allows the IUGR fetus to utilize greater skeletal muscle lactate production to partially offset poor glucose supply via the Cori cycle (Soeters et al., 2021).

#### 2.2.4 Fetal metabolic adaptations: amino acids and protein cycling

The IUGR fetus’s diminished protein supply affects utilization of amino acids for tissue accretion and for energy production. Foundational work by researchers at the University of Colorado School of Medicine found that hindlimb protein accretion was reduced by 55% in IUGR fetal sheep (Rozance et al., 2018; Wai et al., 2018). This coincided with a comparable reduction in total amino acid uptake rates by the hindlimb, although rates among individual amino acids were not affected uniformly (Rozance et al., 2018). For example, hindlimb uptake rates of the essential branched-chain amino acids leucine, valine, and isoleucine, were reduced by up to 73% in IUGR fetal sheep, whereas alanine, glutamine, and glycine were actually secreted from the hindlimb, despite slightly less protein breakdown (Chang et al., 2019a). These differential fluxes are presumably an attempt at metabolic thrift, as branched-chain amino acids can be converted into alanine, glutamine, and glycine, which are substrates for hepatic gluconeogenesis (Chang et al., 2019a). Indeed, expression of enzymes that facilitate this conversion was greater for IUGR fetal rats and sheep (Kloesz et al., 2001; Chang et al., 2019a). Interestingly, long-term infusion of essential amino acids into IUGR fetal sheep did not consistently increase protein accretion, indicating that amino acid utilization was restricted by more than just the short supply (Wai et al., 2018). Together, these studies show definitively that protein accretion in the IUGR fetus is impeded primarily by slower protein synthesis and not by greater protein breakdown. Like amino acid uptake, the impact of IUGR on individual amino acid oxidation rates is not uniform. For example, IUGR fetal sheep oxidized 47% less threonine (Anderson et al., 1997) but 2-fold more lysine (Limesand et al., 2009). Leucine oxidation rates were reduced by 34% in one early study of IUGR fetal sheep (Ross et al., 1996) but were normal in others (Rozance et al., 2018; Wai et al., 2018). Moreover, leucine oxidation was not affected in fetal sheep made chronically hypoxemic (Rozance et al., 2021), hypoglycemic (Carver et al., 1997), hyperglucagonemic (Cilvik et al., 2021), or hyperinsulinemic (Brown et al., 2016) late in gestation, but it was markedly reduced by elevated circulating IGF-1 (Stremming et al., 2021). A greater amount of leucine was oxidized when normal fetuses were experimentally administered excess amino acids (Rozance et al., 2009; Maliszewski et al., 2012), but this effect was blunted in IUGR fetuses (Brown et al., 2012). Amino acids and glucose are normally competitive oxidative substrates (Brown et al., 2017). However, the apparent lack of compensatory amino acid oxidation in IUGR fetuses despite substantial deficits in glucose oxidation may have been the result of mitochondrial deficits. Indeed, IUGR fetal skeletal muscle had less citrate synthase, which is an indicator of intact and functional mitochondria (Stremming et al., 2022). It also expressed less BCAT1 and BCAT2, the transaminase enzymes that convert leucine and isoleucine into the Krebs cycle intermediate succinate, and less ALT, the enzyme that converts alanine to pyruvate (Chang et al., 2019a). The coincident deficits in glucose and amino acid oxidation ultimately diminished ATP content in IUGR fetal muscle (Stremming et al., 2020).

#### 2.2.5 Fetal metabolic adaptations: lipid homeostasis

The impact of IUGR on lipid homeostasis is perhaps less clear. Elevated triglycerides and non-esterified (or free) fatty acids (NEFA) in cord blood at birth is a hallmark of IUGR in humans (Youssef et al., 2021; Chassen et al., 2022) and has been used for decades as a clinical indicator of fetal stress (Elphick et al., 1978). High blood lipid concentrations in late gestation and after birth have also been documented in piglets with spontaneous IUGR (i.e., litter runts) (Li et al., 2018) and in several IUGR sheep models (Wallace et al., 2014; Wallace et al., 2020; Posont et al., 2021; Zhang et al., 2021), although we are aware of at least one study that found *reduced* circulating NEFA in young IUGR-born lambs (Duffield et al., 2009). The impact of IUGR on circulating cholesterol is similarly inconsistent and may depend in part upon the lipoprotein to which cholesterol is bound. For example, Youssef et al. (2021) observed 23% greater total cholesterol but 20% less HDL-C in cord blood from IUGR babies, whereas Pecks et al. (2012) reported reductions in both total cholesterol and HDL-C. Circulating cholesterol was elevated in several IUGR fetal sheep studies and correlated tightly with fetal weights (Meyer et al., 2010; Zywicki et al., 2016; Sun et al., 2018; Zhang et al., 2021). As neonates, however, IUGR-born lambs had normal or even reduced circulating cholesterol, although HDL-C was still elevated (Wallace et al., 2014; Posont et al., 2021). Changes in intracellular lipids within IUGR tissues may also differ between prenatal and postnatal stages. In fetal sheep, for example, IUGR due to nutrient restriction or fetal crowding increased blood NEFA and triglycerides by as much as 50% but reduced liver triglycerides and hepatic lipase, the enzyme responsible for their hydrolysis (Meyer et al., 2010; Zywicki et al., 2016; Sun et al., 2018; Zhang et al., 2021). In IUGR fetal guinea pigs, hepatic utilization of palmitate (the most common saturated fatty acid in animals and plants) was reduced by about 50% (Detmer et al., 1992). Conversely, IUGR-born neonatal goats had greater liver accumulation of NEFA, triglycerides, and cholesterol (Liu et al., 2022), and IUGR-born runt piglets had more expansive hepatic lipid droplets, particularly after undergoing catch-up growth (Wang et al., 2022). IUGR-induced changes in lipid metabolism were largely tissue-specific, as were changes in *de novo* synthesis. Shortly after birth, IUGR infants exhibited greater rates of whole-body triglyceride mobilization and fatty acid oxidation (Patel and Kalhan, 1992). Moreover, gene expression for the key β-oxidation facilitator PPARα was increased in liver tissue from IUGR-born runt piglets (Wang et al., 2022) and in cardiac muscle from IUGR-born adult guinea pigs (Botting et al., 2018). Gene expression for CPT1 and HADHA, which play rate-limiting roles for fatty acid β-oxidation, was reduced in liver tissues of IUGR-born rats (Lane et al., 2001a). However, skeletal muscle from these rats had greater CPT1 and HADHA expression along with greater triglyceride content (Lane et al., 2001b). Cardiac muscle from IUGR fetal sheep also expressed less mRNA for CPT1 and for enzymes associated with β-oxidation and esterification of fatty acids (Drake et al., 2022). This coincided with greater circulating acylcarnitines, which are indicative of impaired or incomplete fatty acid oxidation (Drake et al., 2022). Fatty acid synthesis rates were reduced in liver and lung tissues of IUGR rat fetuses, but not in brain tissue (Vileisis et al., 1982). Interestingly, maternofetal O_2_ supplementation did not recover fatty acid synthesis in these fetuses, which indicates that the deficit was not a product of hypoxemia (Vileisis, 1985). As adults, fatty acid synthesis was normal in liver and muscle from IUGR-born rats but was elevated in adipose tissues (Yee et al., 2016). Disruptions in lipid homeostasis almost certainly contribute to greater adiposity in IUGR-born offspring (Ong et al., 2000; Zinkhan et al., 2018). Indeed, IUGR-born lambs exhibited markedly greater insulin sensitivity for fat deposition (De Blasio et al., 2007b). They also produced muted increases in circulating NEFA in response to epinephrine challenge, indicating a reduced capacity for lipid mobilization (Harwell et al., 1990; Chen et al., 2010).

#### 2.2.6 Fetal metabolic adaptations: insulin secretion

Deficient insulin production and secretion is a key contributor to poor growth and metabolic dysfunction in IUGR fetuses and offspring (Limesand et al., 2006; Xing et al., 2019). Large reductions in basal circulating insulin and complete inhibition of nutrient-stimulated insulin secretion observed in IUGR fetal sheep (Limesand et al., 2007; Cadaret et al., 2019b) were the direct result of fetal hypoxemia and hypoglycemia. To illustrate, when normoxemia and euglycemia were experimentally restored for a 5-day period in IUGR fetal sheep, basal insulin concentrations normalized and glucose-stimulated insulin secretion was rescued (Camacho et al., 2022). Conversely, acute or chronic experimental hypoxemia or hypoglycemia in otherwise uncompromised fetal sheep reduced insulin secretion (Rozance et al., 2006; Yates et al., 2012a; Benjamin et al., 2017). Basal hypoinsulinemia was also resolved postnatal, as IUGR-born offspring were no longer hypoxemic or hypoglycemic after birth (Posont et al., 2021; Cadaret et al., 2022). The suppressive effects of these conditions, particularly hypoxemia, on pancreatic β cell function are mediated in large part by adrenergic signaling. In fact, blocking the adrenergic response to IUGR conditions via pharmaceutical antagonists or adrenal demedullation not only rescued insulin secretion, but in most cases revealed a compensatory enhancement in β cell stimulus-secretion coupling (Leos et al., 2010; Yates et al., 2012a; Macko et al., 2013; Macko et al., 2016). Comparable enhancements in β cell function were observed following chronic norepinephrine infusion into otherwise normal fetal sheep or rats (Chen et al., 2014; Chen et al., 2017; Li et al., 2021). Despite greater sensitivity of β cells, IUGR pancreatic islets are poorly developed. Specific islet deficits are reviewed in detail elsewhere (Boehmer et al., 2017) but include smaller size with fewer β cells/islet, less production and sensitivity to growth factors, and poor vascularity (Limesand et al., 2005; Styrud et al., 2005; Limesand et al., 2006; Ham et al., 2009; Rozance et al., 2015).

### 2.3 Inflammatory contributions to IUGR pathologies

Systemic inflammation has been observed in IUGR fetuses of several species. Although the role of inflammation in IUGR pathologies are not fully characterized, overexposure to inflammatory cytokines is known to disrupt progenitor cell function, tissue growth, and metabolism, as detailed in previous reviews (Joanisse and Parise, 2016; Hicks and Yates, 2021; Most and Yates, 2021). Inflammatory cytokines such as TNFα, IL-1β, and IL-6 were increased in blood and liver of IUGR fetal lambs and mice (Hudalla et al., 2018; Cadaret et al., 2019b; Zhang et al., 2021) and in cord blood of IUGR infants (Amarilyo et al., 2011). In humans, piglets, and rats, circulating inflammatory cytokines remained elevated for the first few hours, days, or even weeks after birth (Krajewski et al., 2014; Riddle et al., 2014; Chisaka et al., 2015; Huang et al., 2019). Eventually, however, high circulating cytokine concentrations subsided and in many cases even fell below normal; plasma TNFα was reduced by 50% in IUGR-born neonatal lambs (Posont et al., 2021), and IFNγ, IL-1β, IL-4, and IL-8 concentrations were reduced in young IUGR-born piglets (Zhong et al., 2012). Resting blood TNFα, IL-6, and IL-1β concentrations were normal in IUGR-born adult rats but were 25%–40% less elevated in response to immune challenge (Desai et al., 2009; Rueda-Clausen et al., 2011). We should note that at least one study of IUGR-born adult rats observed modestly greater circulating inflammatory cytokines (He et al., 2018), although the reason for this unusual finding was not clear. Moreover, when these researchers produced IUGR piglets using the same model of maternal nutrient restriction, they found postnatal circulating cytokine concentrations that were indeed below normal (Dong et al., 2015).

**FIGURE 1:**
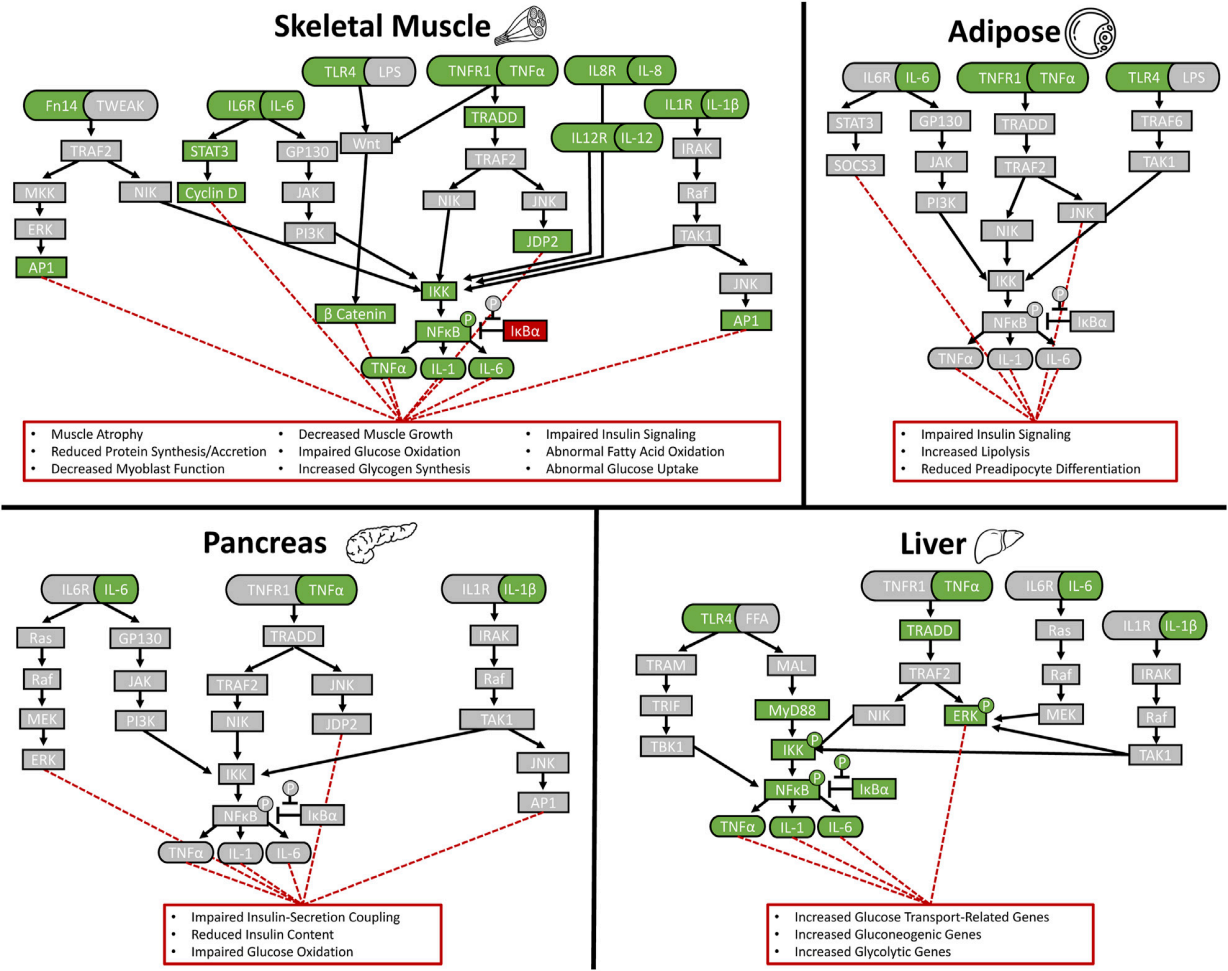
Stress-induced programming of enriched inflammatory signaling pathways in IUGR sheep tissues. Findings compiled from the literature are shown for skeletal muscle (Llovera et al., 1997; Bodell et al., 2009; Gray et al., 2009; Cadaret et al., 2017; Cadaret et al., 2019c; Hicks and Yates, 2021; Posont et al., 2022), liver (Fitzgerald et al., 2003; Zarate et al., 2021; Zhang et al., 2021; Wang et al., 2022), pancreas (Hadjivassiliou et al., 1998; Andersson et al., 2005; Ellingsgaard et al., 2011; Kelly et al., 2017), and adipose tissue (Kras et al., 2000; Sharkey et al., 2009; Beer, 2022). Green boxes indicate signaling components reported to be upregulated and red boxes indicate downregulated components.

Postnatal reductions in circulating inflammatory cytokines are likely a compensatory response to heightened inflammatory sensitivity that develops in several IUGR fetal tissues and persists after birth (Hicks and Yates, 2021). In a landmark example from humans, the soluble form of the TNFα receptor, TNFR1, was observed to be 2.5-fold greater in the umbilical cord of IUGR newborn infants (Laskowska et al., 2007). Enhanced inflammatory sensitivity is also apparent in IUGR skeletal muscle, as hindlimb muscles from IUGR fetal sheep and rats exhibited greater gene expression for TNFR1, the IL-6 receptor (IL6R), and even the TWEAK receptor (Fn14) near term (Cadaret et al., 2019a; Posont et al., 2022). Muscle from these IUGR fetal sheep also contained less of the NFκB arrest protein, IκBα (Posont et al., 2022). As neonates, IUGR-born lambs continued to exhibit greater skeletal muscle TNFR1 content, although IκBα content was increased, perhaps in compensation (Posont et al., 2021). Adaptive enrichment of inflammatory signaling components in the muscle of IUGR-born offspring may arise in part from intrauterine programming of muscle progenitors, which are established prenatal but are incorporated into muscle over the entire lifespan (Allen et al., 1979). Indeed, myoblasts isolated from IUGR fetal sheep had greater gene expression for TNFR1, IL6R, and TLR4 and also had more c-Fos, a cytokine-responsive protein that can disrupt cellular differentiation (Posont et al., 2022). In culture, these IUGR fetal myoblasts exhibited greater phosphorylation of NFκB when stimulated with TNFα and were more sensitive to inhibitors of the canonical signaling enzyme, IκB kinase, under basal conditions (Posont et al., 2022). The impact of enhanced inflammatory sensitivity in skeletal muscle on growth and metabolic homeostasis is substantial, as the tissue comprises about 60% of total juvenile body mass and expands substantially between fetal and juvenile stages (Calnan et al., 2021; Hicks et al., 2021). As illustrated in Figure 1, however, it is not the only tissue to develop this phenotype. Fat tissues of IUGR-born juvenile rats exhibited greater gene and protein expression for TNFα and TNFR1 (Riddle et al., 2014), and transcriptome analyses of pancreatic islets from IUGR fetal sheep indicated enriched TNFα signaling pathways (Kelly et al., 2017). Liver content of TNFα, IL-6, IL-1, TLR4, MyD88, phosphorylated and total NFκB, phosphorylated IκBα, and phosphorylated IκB kinase were elevated in IUGR fetal sheep and newborn mice, although the latter also exhibited tempered hepatic TNFα production in response to acute LPS-stimulation (Zarate et al., 2021; Zhang et al., 2021). Greater hepatic TNFα, IL-6, and TLR4 were also observed in IUGR-born pigs at weaning and in IUGR-born rats in adulthood, which corresponded to greater percentages of phosphorylated IκBα and NFκB (Liu et al., 2014; Tarry-Adkins et al., 2016; He et al., 2018). Intestinal tissues of newborn IUGR piglets exhibited a paradoxical reduction of IFNγ but had increased IL-4 and the inflammation-mediating transcription factor FOXO3a (Zhong et al., 2012). Intestinal tissues from these piglets also exhibited less of the anti-inflammatory cytokine, IL-10 (Zhong et al., 2012). By weaning, intestinal gene and protein expression for cytokines and their canonical pathways were robustly enhanced, which corresponded to greater phospho-activation and nuclear translocation of NFκB and greater activation of the inflammation-mediating kinase, JNK (Niu et al., 2021; Cui et al., 2022). Lung tissue from IUGR-born rats likewise exhibited greater gene expression for inflammatory cytokines and reduced expression for the anti-inflammatory IL-10, which corresponded to greater phospho-activation of the inflammatory mediator, STAT3 (Alejandre Alcazar et al., 2012). Even skin tissues of IUGR-born rats exhibited evidence of enhanced inflammation, as indicated by greater macrophage infiltration and increased gene expression for the interleukin receptor, IL7R, and its cofactor, cytokine receptor-like factor 2 (Polányi et al., 2020). One noteworthy exception for this phenotype appears to be white blood cells, as IUGR-born piglets and lambs had little or no change in circulating leukocyte profiles (Zhong et al., 2012; Amdi et al., 2020; Posont et al., 2021; Cadaret et al., 2022). Moreover, IL-1β production and proliferation by IUGR leukocytes was less responsive to LPS stimulation (Zhong et al., 2012; Amdi et al., 2020).

**FIGURE 2:**
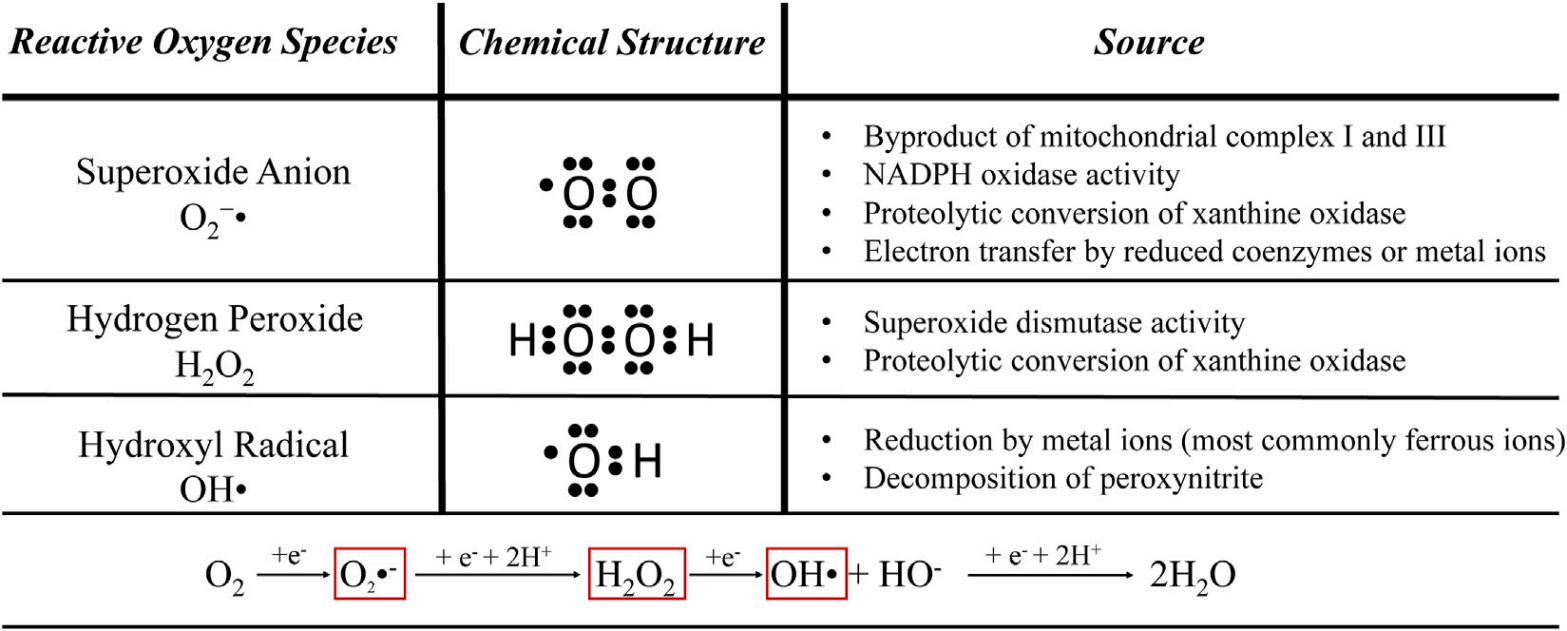
Common stress-induced reactive oxygen species. These are natural cellular byproducts that can be damaging when overproduced during inflammation or other stress conditions (Turrens, 2003; Collin, 2019; Juan et al., 2021).

Inflammation and oxidative stress are inherently-linked stress conditions. The major reactive oxygen species that mediate oxidative stress are listed in Figure 2. These are physiological byproducts that serve important roles in cellular communication, but excessive accumulation causes cellular damage and increases risk for chronic inflammatory diseases (Pizzino et al., 2017). Reactive oxygen species stimulate greater cytokine secretion from many cell types (Bulua et al., 2011; Enoki et al., 2016). They also *directly* stimulate inflammatory pathways by activating IκB kinase, stimulating IκB/NFκB dissociation, and facilitating greater nuclear NFκB dimerization (Lugrin et al., 2014). Coincidentally, inflammatory stimulation often increases production of reactive oxygen species (Floyd et al., 1999; Reid and Li, 2001; Langen et al., 2002; Sriram et al., 2011). For example, skeletal muscle of IUGR-born juvenile rats overexpressed components of NADPH oxidase 2, a membrane-bound enzyme that produces reactive oxygen species for immune signaling but is also a prominent source for overproduction (Chisaka et al., 2015; Tarry-Adkins et al., 2016). The short-lived nature of reactive oxygen species makes them difficult to measure *in situ*, but greater hepatic reduction of oxidized glutathione was indicative of severe oxidative stress in IUGR-born neonatal pigs (Niu et al., 2019). Hepatic concentrations of malondialdehyde and protein carbonyl, the primary indicators of lipid peroxidation and oxidative protein damage, respectively, were also increased in IUGR-born adult rats and neonatal pigs (He et al., 2018; Niu et al., 2019). Tissues of IUGR-born offspring are more susceptible to reactive oxygen species due to maladaptive reductions in several intracellular antioxidant compounds. Hepatic concentrations of the antioxidant enzyme glutathione reductase, which clears oxidized glutathione, was reduced in IUGR-born pigs (Niu et al., 2019). Glutathione reductase was one of several antioxidant proteins for which hepatic gene expression was downregulated in IUGR-born neonatal piglets and rats (Tarry-Adkins et al., 2016; Niu et al., 2019), which culminated in an almost 50% reduction in total antioxidant capacity of the liver (He et al., 2018; Niu et al., 2019).

## 3 Mitigating the impact of IUGR with ω-3 PUFA

### 3.1 Overview of dietary ω-3 PUFA

Nutraceutical use of two natural bioactive ω-3 PUFA, eicosapentaenoic acid (EPA; 20:5 ω-3) and docosahexaenoic acid (DHA; 22:6 ω-3), has increased in popularity as their anti-inflammatory and antioxidant effects have been more firmly established (Shahidi and Ambigaipalan, 2018). Although EPA and DHA can be synthesized *de novo* from α-linolenic acid in modest amounts, additional dietary sources result in health benefits (Russell and Burgin-Maunder, 2012). Many ocean fish and algae species are particularly rich sources of exogenous ω-3 PUFA and are used to produce commercial fish oil-extract supplements (Shahidi and Ambigaipalan, 2018). Consumer interest in ω-3 PUFA has led to the marketing of food products such as milk and bacon that are enriched with exogenous EPA and DHA (Bernal-Santos et al., 2010; Meadus et al., 2010).

Studies performed *in vivo* and *in vitro* report notable anti-inflammatory and antioxidant effects of ω-3 PUFA. Incubation of cultured macrophages with either EPA or DHA for only 48 h mitigated the normally robust LPS-induced secretion of TNFα, IL-1β, and IL-6 by up to 90% (Weldon et al., 2007). This effect was facilitated by stimulation of the GPR120 receptor pathway, which suppresses NFκB binding to DNA and thus inhibits transcription of additional inflammatory factors, including cytokines and toll-like receptors (Weldon et al., 2007; Oh et al., 2010; Block et al., 2012). Even in generally healthy individuals, daily EPA and DHA supplementation reduced circulating IL-6 and TNF-α concentrations by about 15% (Kiecolt-Glaser et al., 2011; Kiecolt-Glaser et al., 2012). In mice genetically engineered to overproduce EPA and DHA, endotoxin-stimulated blood TNFα concentrations were 5-fold less than in normal mice, and hepatic gene expression was reduced for several inflammatory cytokines (Schmocker et al., 2007). Rats supplemented ω-3 PUFA via daily inhalation likewise had less severe elevation of circulating inflammatory cytokines in response to endotoxin (Kocherlakota et al., 2022). Biomedical studies showed that ω-3 PUFA were particularly effective for individuals with chronic inflammatory conditions, including type 2 diabetes, cancers, and cardiovascular diseases (Natto et al., 2019; Xiao et al., 2022; da Silva et al., 2016; Koppelmann et al., 2021). Moreover, DHA and EPA were shown to downregulate production of reactive oxygen species and were effective scavengers of superoxide anions (da Silva et al., 2016; Richard et al., 2008). Indeed, supplementation of ω-3 PUFAs mitigated both systemic inflammation and excessive production of reactive oxygen species in acute and chronic disease states (Li et al., 2017; Alorabi et al., 2022; Gortan Cappellari et al., 2022; Khan et al., 2022). Although few studies have investigated ω-3 PUFA supplementation in IUGR outcomes, extensive literature documenting their antioxidant and anti-inflammatory functions indicate their potential for ameliorating IUGR pathologies.

### 3.2 Inflammation and oxidative stress as targets to improve IUGR outcomes

#### 3.2.1 Potential for recovering growth

Inflammatory cytokines are dynamic regulators of myoblast function and thus play a complex role in postnatal muscle growth (Bodell et al., 2009; Alvarez et al., 2020). *In vitro* studies found that proliferation rates increased when myoblasts were incubated for short periods with low or moderate concentrations of TNFα, IL-1β, or IL-6 (Otis et al., 2014; Alvarez et al., 2020). For TNFα and IL-6 incubations, greater proliferation was coincident with impaired differentiation (Langen et al., 2001; Alvarez et al., 2020; Posont et al., 2022). Indeed, exposure to either cytokine substantially reduced the early differentiation transcription factor, myoD, and modestly reduced the later differentiation factor, myogenin (Alvarez et al., 2020). However, nuclear expression of the myoblast-specific proliferation factor, pax7, was also reduced by these incubations (Alvarez et al., 2020). Furthermore, high physiological concentrations of IL-6 actually diminished myoblast proliferation, and incubation for longer periods of time dampened the inhibitory effect on differentiation (Steyn et al., 2019). These idiosyncratic outcomes were associated with the respective up or downregulation of IL6R by moderate or substantial IL-6 exposure (Steyn et al., 2019). Moreover, IL6R inhibition diminished not only the effects of IL-6 but those of TNFα and IL-1β as well (Alvarez et al., 2020). Nevertheless, all cytokine effects were lost when myoblasts were incubated with NFκB inhibitors (Otis et al., 2014). Disruption of the balance between myoblast proliferation and differentiation clearly reduces the capacity for muscle hypertrophy. To illustrate, intramuscular IL-6 infusion in young rats decreased muscle growth by about 10% over 2 weeks (Bodell et al., 2009). This coincided with greater gene expression for cytokine signaling components, including TNFα, TNFR1, and atrogin-1 (Haddad et al., 2005; Bodell et al., 2009). In TNFα-infused mice, an observed increase in muscle catabolism was attributed to greater ubiquitin-dependent proteolysis (Llovera et al., 1997). The combination of greater relative muscle catabolism and decreased myoblast-facilitated muscle hypertrophy associated with experimentally-elevated cytokine exposure was comparable to the phenotype observed in the IUGR fetus (Yates et al., 2014; Soto et al., 2017; Rozance et al., 2018; Posont et al., 2022).

Like inflammation, oxidative stress impairs skeletal muscle hypertrophy in similar but independent fashion (Jang et al., 2020). Addition of even modest concentrations of reactive oxygen species to myoblast incubations increased proliferation (Sciancalepore et al., 2012) and inhibited differentiation (Langen et al., 2002; Sandiford et al., 2014), regardless of the presence of inflammatory cytokines. When primary mouse myoblasts were engineered to overproduce H_2_O_2_, only about half as many stained positive for myogenin, expressed myosin heavy chain protein, or fused with other myoblasts (Sandiford et al., 2014). Furthermore, accumulation of reactive oxygen species both directly and indirectly resulted in muscle catabolism (Jang et al., 2020). In addition to directly damaging cellular proteins, elevated reactive oxygen species in antioxidant-deficient mice upregulated cysteine proteases, which increased protein breakdown by the ubiquitin-proteasome and autophagy-lysosome systems (Muller et al., 2006; Jang et al., 2020). These mice were characterized by a reduction in muscle mass of up to 50%, along with poor exercise performance (Muller et al., 2006).

#### 3.2.2 Potential for improving metabolism

Sustained exposure to elevated inflammatory cytokines or reactive oxygen species dysregulates skeletal muscle glucose metabolism and impairs insulin signaling and secretion. Brief incubations with high physiological IL-6 concentrations increased glucose uptake, oxidation, and incorporation into glycogen and also reduced lactate production in primary human and rodent muscle, as well as in culture-derived myotubes (Al-Khalili et al., 2006; Glund et al., 2007; Gray et al., 2009; Cadaret et al., 2017). Glucose uptake was stimulated at much lower IL-6 concentrations when its receptor was added to the incubation in concert (Gray et al., 2009). In primary rat muscle and myotubes, TNFα similarly elevated glucose uptake and oxidation (Yamasaki et al., 1996; Cadaret et al., 2017). Moreover, greater glucose oxidation was observed *in vivo* when healthy young adults were infused with moderate amounts of IL-6 over several hours (Carey et al., 2006). Some of these studies indicated that cytokine regulation of muscle glucose utilization was independent of insulin and its major pathway components, IRS1 and Akt (Yamasaki et al., 1996; Glund et al., 2007; Gray et al., 2009; Cadaret et al., 2017). Rather, cytokines appeared to stimulate translocation of sequestered Glut4 glucose transporters to the membrane by activating the kinase enzyme, AMPK (Al-Khalili et al., 2006; Carey et al., 2006). In fact, longer periods of cytokine exposure generally disrupted insulin signaling (Rotter et al., 2003; Al-Khalili et al., 2006; Cadaret et al., 2017), although the direct effects on glucose utilization remained. This was demonstrated particularly well by *in vitro* incubation of myoblast cell lines with TNFα, which increased basal glucose uptake by almost 3-fold but reduced insulin-stimulated glucose uptake by about 75% (Yamasaki et al., 1996; Grzelkowska-Kowalczyk and Wieteska-Skrzeczynska, 2006; Remels et al., 2015). Infusion of TNFα into mice resulted in a 50% reduction of insulin-stimulated hindlimb glucose uptake but did not affect resting glucose uptake rates (Youd et al., 2000). The primary target of cytokines within the insulin signaling pathway appears to be the phospho-activation of Akt, a hub that facilitates myriad insulin effects (Ji et al., 2022). Incubation of primary rat muscle with moderate concentrations of TNFα or IL-6 for as little as 2 h reduced insulin-stimulated Akt phosphorylation by more than 50% (Cadaret et al., 2017). Similar deficits were observed in culture-derived myotubes after 6-day incubation with TNFα (Grzelkowska-Kowalczyk and Wieteska-Skrzeczynska, 2006) and in primary bovine adipocytes after 12 h (Du et al., 2022). In addition to disrupting insulin signaling, cytokines also impair insulin stimulus-secretion coupling in pancreatic islets, although this effect is quite dependent upon duration of exposure. In fact, when mice were injected with IL-6, glucose-stimulated insulin secretion was *increased* over the first 45 min, but this effect was gone by 1 h (Ellingsgaard et al., 2011). Incubation of primary mouse islets with IL-1β alone or in combination with IFNγ increased glucose-stimulated insulin secretion for the first few hours but reduced it after 20 h (Andersson et al., 2005). Likewise, 48-h incubation with TNFα alone or together with IL-1β and IFNγ had no effect on basal insulin secretion but completely inhibited glucose-stimulated insulin secretion in primary rat islets and in INS-1 rat β cells (Hadjivassiliou et al., 1998; Zhang et al., 2022). This combination of cytokines also reduced insulin content in primary human islets after 48 h (Hadjivassiliou et al., 1998). The 40% reduction in glucose-stimulated insulin secretion and 25% reduction in pyruvate-stimulated insulin secretion observed in INS-1 rat β cells after 24 h with IL-1β and IFNγ coincided with reduced indicators of oxidative metabolism, the facilitating mechanism for β cell stimulus-secretion coupling (Rozance et al., 2007; Barlow et al., 2018).

Like cytokines, *in vitro* exposure of primary rat muscle to reactive oxygen species for less than 1 h increased glucose uptake by up to 48% and glycogen synthase activity by about 20% by enhancing canonical insulin signaling (Kim et al., 2006; Higaki et al., 2008). In fact, concurrent exposure to insulin and reactive oxygen species for 20 min had an additive effect on glucose uptake (Higaki et al., 2008). Short-term oxidative stress reflects the normal microenvironment of muscle and is not particularly harmful, but sustained exposure becomes deleterious (Higaki et al., 2008; Diamond-Stanic et al., 2011). Incubation of rat muscle with high concentrations of reactive oxygen species ceased stimulating basal glucose uptake after 6 h (Diamond-Stanic et al., 2011). Insulin-stimulated glucose uptake and Akt phosphorylation were hindered by reactive oxygen species after only 2 h (Diamond-Stanic et al., 2011). The adverse effects of longer exposure on insulin signaling components were mediated in part by p38 MAPK and were evident even at modest reactive oxygen species concentrations (Archuleta et al., 2009; Diamond-Stanic et al., 2011). Interestingly, reactive oxygen species did not appear to affect function of primary rat or human islets after 48 h of incubation (Hadjivassiliou et al., 1998).

Inflammatory cytokines help to regulate lipid homeostasis and metabolism by skeletal muscle in coordination with their effects on muscle glucose utilization. Infusion of IL-6 into healthy individuals for 3 hours increased total fatty acid oxidation rates during and for several hours after the infusion period (van Hall et al., 2003). In primary rat muscle, 1-h incubations with high IL-6 concentrations increased palmitate oxidation but not deposition, whereas 1-h incubation with TNFα increased deposition but not oxidation (Bruce and Dyck, 2004). Conversely, 3-h incubation of culture-derived human myotubes with IL-6 increased palmitate uptake and oxidation rates (Al-Khalili et al., 2006). Greater oxidation occurred in the presence and absence of insulin and was mediated primarily by AMPK pathways (Bruce and Dyck, 2004; Al-Khalili et al., 2006). To facilitate greater lipid utilization by muscle, cytokines concurrently mobilize lipid deposits from adipose and other tissues. This was demonstrated by infusions of moderate or high concentrations of IL-6 into humans, both of which elevated circulating NEFA and triglycerides (van Hall et al., 2003). Moreover, blocking IL-6 signaling by administering a long-lasting IL6R antagonist reduced indicators of fatty acid mobilization in adult men during various degrees of physical activity and across a range of body mass indices (Trinh et al., 2021). In primary rat muscle, IL-6 stimulated lipid mobilization in part by disrupting insulin’s lipogenic effects (Bruce and Dyck, 2004). Unlike IL-6, TNFα had no impact on endogenous or exogenous skeletal muscle fatty acid oxidation (Bruce and Dyck, 2004). However, several *in vitro* studies have shown that even modestly elevated TNFα concentrations stimulated lipolysis (Ryden et al., 2004; Lee and Fried, 2012; Du et al., 2022). This was recapitulated by 7-day TNFα infusion into mice, which elevated their circulating triglycerides and NEFA concentrations (Li L. et al., 2009). Moreover, both glycerol and NEFA were released from adipocytes at greater rates when stimulated with TNFα *in vitro* (Green et al., 2004; Ryden et al., 2004). TNFα-mediated lipolysis was facilitated in large part by downregulation of perilipin, a protein that mediates hormone-sensitive lipase activity in lipid droplets (Morin et al., 1995; Wu et al., 2004). Studies in rats and cultured adipocytes found that TNFα also reduced activity and expression of lipoprotein lipase, an enzyme that breaks down circulating lipoproteins for deposition into adipocytes (Morin et al., 1995; Wu et al., 2004). The increased production of reactive oxygen species in cytokine-stimulated adipocytes further contributed to lipolysis (García et al., 2021), an effect that was dampened by concurrent inhibition of superoxide production in culture (Krawczyk et al., 2012; Issa et al., 2018). The impact of reactive oxygen species on adipocyte lipolysis coincided with phospho-activation of hormone sensitive lipase (Krawczyk et al., 2012; Issa et al., 2018).

### 3.3 Prenatal and perinatal supplementation of ω-3 PUFA

#### 3.3.1 Potential benefits

Benefits of ω-3 PUFA supplementation during critical windows for fetal development on growth and metabolic outcomes have been documented in humans and animals. Meta-analyses of global health records and clinical trials indicated that dietary ω-3 PUFA supplementation over the 2nd half of gestation was associated with reductions of up to 73% in perinatal mortality rates as well as fewer neonatal intensive care stays (Saccone et al., 2015; Saccone et al., 2016; Ali et al., 2017; Middleton et al., 2018). The supplements were also associated with lower incidence of IUGR in many world populations (Cetin et al., 2002; Agostoni et al., 2005; Fares et al., 2015; Saccone et al., 2015; Gholami et al., 2020). In one prime example, DHA supplementation in *primigravidae* women in Mexico reduced IUGR rates by half (Ramakrishnan et al., 2010). Better pregnancy outcomes coincided with positive effects on uteroplacental tissues, as clinical trials found that ω-3 PUFA supplements increased uterine and umbilical blood flow and reduced placental apoptosis (Wietrak et al., 2015; Ali et al., 2017). Moreover, dietary ω-3 PUFA supplementation in pregnant rats reduced indicators of placental oxidative stress and increased placental and fetal mass (Jones et al., 2013). Even direct infusion of EPA into the bloodstream of IUGR fetal sheep benefitted placental function, as maternofetal glucose and O_2_ gradients were improved (Beer et al., 2021). Importantly, maternal ω-3 PUFA supplements directly benefit the fetus as well as the placenta. Clinical trials found that IUGR fetuses and newborns were deficient in ω-3 PUFA due to impaired *de novo* production and that maternal supplementation was effective in increasing circulating concentrations in the fetus/newborn (Cetin et al., 2002; Agostoni et al., 2005; Llanos et al., 2005; Fares et al., 2015; Wietrak et al., 2015). These findings were recapitulated in rats, where IUGR-born neonates were found to be DHA-deficient, but maternal supplementation increased circulating DHA concentrations in these offspring by 3.25-fold (Morand et al., 1981; Joss-Moore et al., 2010). In pigs, maternal ω-3 PUFA supplementation increased DHA and EPA and reduced ω-6:ω-3 PUFA ratios in fetal blood and skeletal muscle near term, which coincided with greater blood glucose, less cholesterol, and a reduction in the incidence of IUGR from 22% to 14% (Heras-Molina et al., 2021; Heras-Molina et al., 2022). These benefits persisted after birth, as weaning-aged pigs born to ω-3 PUFA-supplemented sows had less circulating total cholesterol, HDL-C, and LDL-C as well as greater total ω-3 PUFA content and lower ω-6:ω-3 PUFA ratios in adipose, muscle, and liver tissues (Heras-Molina et al., 2020). When pregnant rodents carrying IUGR pregnancies were supplemented DHA, birthweight and neonatal growth of their offspring were improved without increased adiposity (Bagley et al., 2013; Velten et al., 2014). Offspring from dams supplemented DHA or ω-3 PUFA-rich fish oil had greater circulating adiponectin, smaller adipocytes, and adipose tissue that expressed more PPARγ, adiponectin, and adiponectin receptors R1 and R2 (Bringhenti et al., 2011; Bagley et al., 2013; Ghnaimawi et al., 2022). Supplementing pregnant cows with ω-3 PUFA (delivered as Ca^2+^ salts for ruminal protection) during late gestation increased circulating DHA in the dam by 5-fold, and calves born to supplemented cows exhibited 6%–10% better average daily gain in the feedlot (Marques et al., 2017). At harvest, these calves produced marginally larger carcasses and loin muscles with ∼10% greater marbling (Marques et al., 2017). In sheep, maternal supplementation of ω-3 PUFA Ca^2+^ salts for the final trimester of gestation had no adverse effects on dam or fetus and increased blood concentrations of EPA and DHA in the ewe by ∼40% and in the newborn lamb by ∼50% (Coleman et al., 2018; Rosa Velazquez et al., 2020; Rosa-Velazquez et al., 2022). Greater fetal ω-3 PUFA availability appeared to be particularly beneficial to IUGR muscle and pancreatic islets. In mice, protein content for the insulin receptor and the myogenic transcription factor, myoD, as well as several genes regulating myogenesis were upregulated in skeletal muscle of newborn pups born to ω-3 PUFA-supplemented dams (Ghnaimawi et al., 2022). Moreover, 5-day infusion of EPA directly into IUGR fetal sheep improved growth of several muscles, restored normal fiber type proportions in the *semitendinosus*, and modestly improved deficits in muscle glucose uptake and oxidation (Lacey, 2021; Lacey et al., 2021). It also recovered about 50% of the deficit in glucose-stimulated insulin secretion (Lacey, 2021; Lacey et al., 2021). These benefits coincided with anti-inflammatory indicators, as EPA infusion ameliorated the elevated circulating TNFα concentrations and reduced total circulating white blood cells observed in IUGR fetal sheep (Lacey, 2021; Lacey et al., 2021). Likewise, maternal ω-3 PUFA supplementation reduced oxidative stress in brain tissues of fetal rats and resolved the heightened macrophage invasion observed in lung tissues of IUGR newborn mice pups (Velten et al., 2014).

Postnatal supplementation of ω-3 PUFA to IUGR-born offspring is also effective in improving metabolic deficits. For example, IUGR-born rats exhibited 50% reductions in circulating triglycerides and 33% reductions in blood urea nitrogen when nursing foster dams that were fed diets high in ω-3 PUFA (i.e., supplemented to offspring via milk) (Voggel et al., 2022). Likewise, directly feeding ω-3 PUFA-enriched diets or supplementing ω-3 PUFA-rich fish oil to IUGR-born juvenile rats partially resolved their hyperlipidemia and reduced their adiposity (Wyrwoll et al., 2006; Bringhenti et al., 2011; Chen et al., 2016; Chicco et al., 2016). It also decreased adipocyte size and leptin secretion, ameliorated inflammatory markers in adipose tissues, and resolved elevated TNFα in circulation (Wyrwoll et al., 2006; Bringhenti et al., 2011; Mark et al., 2014). Postnatal fish oil supplementation improved HOMA-estimated insulin sensitivity, glucose tolerance, and markers of systemic inflammation in IUGR-born rats (Bringhenti et al., 2011; Chen et al., 2016).

#### 3.3.2 Potential limitations

As of this writing, fish oil and other ω-3 PUFA supplements are considered safe for pregnant women by the American Pregnancy Association, which is supported by several Cochrane meta-analyses (Kar et al., 2016; Middleton et al., 2018; Wieland, 2019). Challenges regarding the use of ω-3 PUFA as dietary supplements in livestock include post-ingestive feedback (Freidin et al., 2011), bioavailability in ruminants (Chikunya et al., 2004), and meat sensory traits when fed at high concentrations (Burnett et al., 2020). Reduced palatability and ruminal microbiome changes associated with fish oil can cause animals to eat less when too much is included in a dietary ration, and livestock studies have indicated that the threshold limit is around 2% of the diet. Indeed, inclusion of fish oil at 1% of the diet in dairy cows did not affect *ad libitum* intake, but inclusion at 2% and 3% reduced intake by up to 11% and 32%, respectively (Donovan et al., 2000; Whitlock et al., 2002; Alizadeh et al., 2012; Kairenius et al., 2018). Although milk yield was increased by dietary fish oil at 1% and 2% in these cows, the large drop in dietary intake at 3% caused milk yield to fall (Donovan et al., 2000). Calves fed milk replacer enriched with 0.5% fish oil maintained their intake from birth to 2 months of age, which facilitated a modest increase in their average daily gain (Melendez et al., 2022). Lambs fed Ca^2+^ salts of ω-3 PUFA at 1.5% also maintained dry matter intake (Carranza Martin et al., 2018). In Angus feedlot steers, dry matter intake was reduced by dietary inclusion of fish oil at 2.4% but not at 0.8% or 1.6% (Shingfield et al., 2010). Limits on dietary inclusion could be problematic for ruminant livestock, as bioavailability of ω-3 PUFA is reduced by the markedly high rates of microbial biohydrogenation (i.e., saturation of previously unsaturated fatty acids) in the rumen (Jenkins et al., 2008). To illustrate, 93% of EPA and DHA from unprotected sources were absorbed unmodified across the intestinal wall of the rat (Chen et al., 1985), but as little as 7% were absorbed unmodified in cows (Dewhurst and Moloney, 2013). Similar outcomes were created by the rumen microbiome in sheep, where biohydrogenation rates were upward of 75% (Chikunya et al., 2004; Jenkins et al., 2008). Processing techniques for ω-3 PUFA-rich feed ingredients like flaxseed increased bioavailability of EPA by 30% and total ω-3 PUFA by 23% in lambs (Kronberg et al., 2012). In young goats, heat treatment of linseed oil diets increased DHA bioavailability by 62% and total ω-3 PUFA bioavailability by 19%, which corresponded with greater liver and adipose tissue concentrations (Wang et al., 2019). Freeze-drying of a microalgae-based supplement reduced ruminal biohydrogenation of EPA in lambs from 80% to about 45% (Vítor et al., 2021). Supplements utilizing Ca^2+^ salts of ω-3 PUFA helped maintain or increase bioavailability through ruminal passage without producing off-target effects such as reduced intake and milk fat (Castañeda-Gutiérrez et al., 2007; Oyebade et al., 2020). It is worth noting that even unprotected dietary sources still deliver meaningful amounts of absorbable ω-3 PUFA in ruminants. For example, inclusion of 1.1% fish oil in a silage-based diet for dairy cows increased the amount of EPA and DHA bypassing the rumen microbiome by 2-fold and 3-fold, respectively, despite 78% and 83% biohydrogenation rates (Kairenius et al., 2018). In meat animals, ω-3 PUFA supplementation strategies must consider the effects on product appearance and shelf life. Like all fatty acids, ω-3 PUFA are prone to oxidation that can discolor the surface of meat products (Burnett et al., 2020). Steers fed diets with 3% fish oil had 1.5-fold–2-fold greater carcass EPA and DHA content at harvest (Vatansever et al., 2000). Hamburger patties from these steers reached unacceptable discoloration 1–3 days sooner than normal and cooked sirloin scored about 11% lower in *overall liking*, despite no reduction in flavor score (Vatansever et al., 2000). It should be noted that most of the individual fatty acids measured in this study were increased in meat from fish oil-supplemented animals and that linseed oil supplementation in the same study increased ω-3 PUFA content without the same adverse sensory effects. Thus, adverse sensory traits may be attributable more to the use of a high volume of fish oil as a source than to the increase in ω-3 PUFA *per se*. Indeed, palatability and tolerability of German sausage products were not affected by enrichment with ∼1% purified EPA, DHA, and α-linolenic acid (Köhler et al., 2017). Nevertheless, fish products are the most common source for ω-3 PUFA supplements in livestock (Burnett et al., 2020), and lamb cuts from animals fed diets with 9% fish meal for 6 weeks were scored ∼9% lower in *juiciness* by a trained sensory panel, although flavor, aroma, and palatability were not affected (Ponnampalam et al., 2002). Likewise, meat from lambs fed diets enriched with 1.5% fish oil for 6 weeks was scored 14% lower in overall palatability, despite no reduction in individual scores for flavor, aroma, or juiciness and no issues with discoloration or shelf life (Ponnampalam et al., 2001; Ponnampalam et al., 2002). Because of the potential for reduced meat sensory traits, investigation into the benefit of withdrawing ω-3 PUFA supplements prior to harvest may be warranted.

## 4 Summary

Fetal IUGR is a common condition in humans and animals that impacts metabolic function and growth capacity before and after birth. It is most often associated with prenatal stressors during the critical window for placental development, which functionally and structurally stunts placental tissues. Placental insufficiency limits O_2_ and nutrient delivery to the growing fetus, which by late gestation can no longer keep up with fetal requirements for growth. Consequently, the fetus undergoes a prolonged period of progressive hypoxemia and hypoglycemia that in turn increases fetal adrenergic and inflammatory tones and induces oxidative stress. These changes drive nutrient-sparing adaptations, which manifest in asymmetric fetal growth restriction and thrifty metabolic function that persist postnatal. Mechanisms for these adaptations include reduced adrenergic responsiveness, enhanced inflammatory sensitivity, and increased oxidative stress. IUGR adaptations aid fetal survival, but after birth they increase health risks in humans and reduce growth efficiency in livestock. Enhanced inflammatory sensitivity in IUGR skeletal muscle and other tissues appears to be a key underlying factor in growth and metabolic pathologies. Consequently, anti-inflammatory nutraceuticals such as ω-3 PUFA have yielded promise as potential supplemental strategies for recovering health and performance outcomes in IUGR fetuses and offspring. However, several potential factors must be considered, including issues of palatability, bioavailability, and potential off-target effects. Nevertheless, initial proof-of-concept studies indicate that dietary ω-3 PUFA supplements could provide the basis for recovering growth and metabolic function in IUGR-born individuals.
